# Fusiform Gyrus Dysfunction is Associated with Perceptual Processing Efficiency to Emotional Faces in Adolescent Depression: A Model-Based Approach

**DOI:** 10.3389/fpsyg.2016.00040

**Published:** 2016-02-01

**Authors:** Tiffany C. Ho, Shunan Zhang, Matthew D. Sacchet, Helen Weng, Colm G. Connolly, Eva Henje Blom, Laura K. M. Han, Nisreen O. Mobayed, Tony T. Yang

**Affiliations:** ^1^Department of Psychiatry, University of California, San Francisco, San FranciscoCA, USA; ^2^Cognitive Science, University of California, San Diego, La JollaCA, USA; ^3^Neuroscience Graduate Program and Department of Psychology, Stanford University, Palo AltoCA, USA; ^4^The Osher Center for Integrative Medicine, University of California, San Francisco, San FranciscoCA, USA; ^5^Clinical Neuroscience, Karolinska InstitutetStockhold, Sweden

**Keywords:** adolescent, depression, fMRI BOLD, response time modeling, mood disorders, face processing, fusiform gyrus

## Abstract

While the extant literature has focused on major depressive disorder (MDD) as being characterized by abnormalities in processing affective stimuli (e.g., facial expressions), little is known regarding which specific aspects of cognition influence the evaluation of affective stimuli, and what are the underlying neural correlates. To investigate these issues, we assessed 26 adolescents diagnosed with MDD and 37 well-matched healthy controls (HCL) who completed an emotion identification task of dynamically morphing faces during functional magnetic resonance imaging (fMRI). We analyzed the behavioral data using a sequential sampling model of response time (RT) commonly used to elucidate aspects of cognition in binary perceptual decision making tasks: the Linear Ballistic Accumulator (LBA) model. Using a hierarchical Bayesian estimation method, we obtained group-level and individual-level estimates of LBA parameters on the facial emotion identification task. While the MDD and HCL groups did not differ in mean RT, accuracy, or group-level estimates of perceptual processing efficiency (i.e., drift rate parameter of the LBA), the MDD group showed significantly reduced responses in left fusiform gyrus compared to the HCL group during the facial emotion identification task. Furthermore, within the MDD group, fMRI signal in the left fusiform gyrus during affective face processing was significantly associated with greater individual-level estimates of perceptual processing efficiency. Our results therefore suggest that affective processing biases in adolescents with MDD are characterized by greater perceptual processing efficiency of affective visual information in sensory brain regions responsible for the early processing of visual information. The theoretical, methodological, and clinical implications of our results are discussed.

## Introduction

Major depressive disorder (MDD) is a prevalent condition that is associated negative mood and emotional dysregulation, with an onset that increases dramatically during adolescence (Merikangas et al., 2009; Kessler et al., 2010). While adolescence is both a period of increased brain plasticity and heightened risk for the development of MDD, the neural mechanisms underlying adolescent MDD are still unclear (Casey et al., 2008; Kerestes et al., 2014). Prior research examining the neurobiological mechanisms of individuals with MDD have used facial emotion processing tasks in conjunction with functional magnetic resonance imaging (fMRI) to probe how MDD is related to neural systems supporting affective processing (Fusar-Poli et al., 2009; Stuhrmann et al., 2011). In these fMRI studies of facial emotion processing, adults with MDD compared to healthy controls exhibit brain activation differences at multiple levels in the information processing: from visual areas such as the fusiform gyrus and the middle occipital cortex involved in early visual processing of affective stimuli, to limbic and paralimbic regions such as the amygdala and insula involved in evaluating and integrating sensory and affective information, to prefrontal areas such as dorsolateral prefrontal cortex and ventromedial prefrontal cortex involved in top-down emotion regulation (Haxby et al., 2000; Stuhrmann et al., 2011). More recent work in adolescents with MDD (Ho et al., 2014, 2015; Henje Blom et al., 2015) have also shown concordance with the adult literature by demonstrating that depression is related to functional aberrations in the face processing network that includes visual regions, limbic and paralimbic structures, and frontal cortices during processing of emotional facial expressions in youth.

The most common cognitive feature of MDD is a processing bias toward negatively affective stimuli (Gotlib et al., 2004; Foland-Ross and Gotlib, 2012). The literature also supports conceptual models positing that biases in the processing and interpretation of emotional facial expressions as social cues may be one of the underlying mechanisms in the development of MDD in youth (Joormann and Gotlib, 2006; Joormann et al., 2007; Kujawa et al., 2011). Specifically, attentional biases have been reported even in children who are at familial risk for MDD, thus potentially serving as a cognitive risk factor in the development of depression (Joormann and Gotlib, 2006; Joormann et al., 2007; Kujawa et al., 2011; Montagner et al., 2015). However, whether depression-related biases in the processing of emotional facial expressions are due to dysfunction in early visual regions, limbic and paralimbic regions involved in the affective evaluation of facial expressions, and/or top–down cognitive control regions still remains unclear. Thus, relating biases in the processing of emotional information to neural substrates in adolescents with MDD is critical if we are to understand how these cognitive processes may contribute to the early development of depressive symptoms.

Most cognitive tasks used to assess information processing in both healthy and clinical populations involve straightforward two-choice decisions (e.g., “Is this face negative or positive?”, “Is this word threatening or not?”, “Have you seen this image before or not?”). The behavioral data acquired from these tasks are typically reported as mean response time (RT) and mean accuracy. While comparing effects of RT and accuracy are sometimes meaningful, there are several situations where comparisons of mean RTs or accuracy rates do not sufficiently identify processing differences between groups or conditions (including but not limited to speed-accuracy tradeoffs or an unequal weighing of decision outcomes; (White et al., 2010). Moreover, other behavioral performance measures, such as d’ from signal detection theory, do not take into account RT distributions, and rely only on hits and false alarm rates to explain behavior (Ratcliff and McKoon, 2008; White et al., 2010). Consequently, merely analyzing mean RT and accuracy rates glosses over the potentially complex relationship between RT, accuracy, and the underlying cognitive processes.

Over the past several decades, a variety of mathematical models of choice behavior have successfully related the shape of correct and incorrect RT distributions with the probabilities of making correct or incorrect judgments (Ratcliff and Smith, 2004; Smith and Ratcliff, 2004). The advantage of these models over traditional analyses of accuracy and RT is that both accuracy and RT are used to decompose the behavioral data into distinct information processing components, which are represented in the model as individual parameters. Thus, the model can be fit to behavioral data to separate out and compare distinct decision components, including: perceptual processing efficiency, response caution, response bias, or non-decision time. Sequential sampling models of choice behavior can identify different decision components because they utilize *all* of the behavioral data available (e.g., hits, false alarms, and RT distributions for correct and error responses).

While such models have been used extensively in the field of cognitive psychology, they have only been applied recently to clinical data (White et al., 2010; Pe et al., 2013; Ho et al., 2014; Weigard and Huang-Pollock, 2014). For example, Pe et al. (2013) recently used a sequential sampling model to show that rumination accounts for the attentional bias toward emotionally negative stimuli in adults with MDD (Pe et al., 2013). Their results revealed that when focusing on a negative target, both rumination and depression were associated with facilitated perceptual processing due to negative distracters, whereas only rumination was associated with less interference by positive distracters. Importantly, these results were *not* reproduced when using only accuracy scores or average RTs. Thus, such models possess great potential in allowing researchers to identify the cognitive loci of processing differences between healthy and clinical populations. When combined with neuroimaging, mathematical models of choice behavior can be used to link conceptual processes to neural substrates, thereby providing an unprecedented advance in relating brain dynamics to behavior, symptoms, and functioning.

In the present study, we sought to apply a mathematical model of choice behavior to a sample of acutely depressed and well-matched healthy adolescents undergoing fMRI during a two-choice facial emotion identification task. All subjects completed an emotion identification task of dynamically morphing faces that has been demonstrated to robustly activate frontolimbic regions implicated in the pathophysiology of adolescent MDD (Ho et al., 2015). One of the key parameters examined in sequential sampling models of choice behavior is the *drift rate*, which indexes the strength or amount of sensory information for a particular choice option (i.e., “sensory evidence”), and thus, acts as a proxy for perceptual processing efficiency in conditions when signal and noise amounts do not differ. Drift rates are often the primary focus of researchers employing two-choice tasks as they provide a more direct index of perceptual processing efficiency than either RTs or accuracy, as the latter two measures are affected by the other components of the decision process. In the context of a mathematical model of choice behavior, the effects of the decision components not related to the drift rate are parsed out and represented as other parameters in the model. Since these components are separated they do not affect drift rate estimates despite obviously influencing RT and accuracy. Thus, drift rates are better able to detect small differences in perceptual processing efficiency that might not be as readily captured by simply comparing RTs or accuracy (White et al., 2010). Given that prior studies have found that drift rates differ in adolescents and adults with MDD during emotional and cognitive processing (Pe et al., 2013; Ho et al., 2014), we focused our analyses on the drift rate parameter.

The present study is a secondary analysis of a prior fMRI investigation where we compared adolescents with MDD and healthy controls on a facial emotion identification task (Ho et al., 2015). However, in our previous investigation we examined only task-based versus resting-state functional connectivity of the medial prefrontal cortex and posterior cingulate cortex, two hubs of a major task-negative network. Thus, the hypotheses in our previous investigation centered only on this task-negative network and included both task-based and resting-state functional connectivity analyses. The present study differs considerably from our previous investigation in that our goal is to use a combination of neuroimaging and mathematical models of choice behavior to investigate potential neural correlates of affective processing biases in adolescent MDD. The whole-brain fMRI results that we report here and the results of our mathematical model on the behavioral data in this task are therefore novel. Thus, the primary contribution of the present study is to demonstrate the utility of applying mathematical models of choice behavior to investigate cognitive processing differences in a clinical sample of depressed adolescents compared to healthy controls, and to relate these behavioral assessments with functional neuroimaging measures.

The brain regions we hypothesized would differ between adolescents with MDD and healthy controls include the face processing network, specifically occipital areas, limbic and paralimbic structures, and prefrontal regions. Because the drift rate parameter in the LBA model captures perceptual processing efficiency to the visual stimuli in our task, we also hypothesized that depression-related task activation in visual processing regions (e.g., fusiforym gyrus, middle occipital cortex) will correlate with drift rate in adolescents with MDD. In the following sections, we describe the experiment and analyses and conclude with a discussion on the implications of this work and future directions.

## Materials and Methods

### Participants

Sociodemographic, clinical, neuroimaging and behavioral data from a total of 63 adolescents were included in this study. This sample of 63 adolescents has been described previously (Ho et al., 2015). To briefly summarize, 26 adolescents (7 males, mean ± SEM age: 16.1 ± 0.3 years) were diagnosed with a current episode of MDD and 37 (14 males, mean ± SEM age: 16.0 ± 0.2 years) HCL. Potential MDD participants were recruited from adolescent psychiatric and primary care clinics in San Diego, while potential HCL participants were recruited from the same geographic area via e-mail, internet, or flyers. Adolescents from both genders and all ethnicities were allowed to participate and all subjects received financial compensation for participating in this study. All participating adolescents provided written informed assent and their parent(s)/legal guardian(s) provided written informed consent in accordance with the Declaration of Helsinki. The Institutional Review Boards at the University of California, San Diego, University of California, San Francisco, Rady Children’s Hospital in San Diego, and the County of San Diego approved this study. All participants received financial compensation.

The Schedule for Affective Disorders and Schizophrenia for School-Age Children-Present and Lifetime Version (K-SADS-PL; Kaufman et al., 2000) was administered to all potentially depressed adolescents. All depressed adolescents in the study met full criteria for a current primary diagnosis of MDD according to *DSM-IV* and were unmedicated at the time of scanning (all depressed participants were entirely naïve to antidepressants except for two: one had last been exposed to antidepressants 4 months before their scan and other, 4 years before their scan). The computerized Diagnostic Interview Schedule for Children 4.0 (Shaffer et al., 2000) and the Diagnostic Predictive Scale (Lucas et al., 2001) were used to screen for the presence of any Axis I diagnoses in the HCL adolescents. The K-SADS-PL was administered by mental health professionals with prior clinical experience with children and adolescents (e.g., child and adolescent psychiatrists or psychologists) and research assistants who were rigorously trained in order to develop a high standard of proficiency. All of the K-SADS-PL interviewers were trained to a kappa level of 0.80 or higher for the diagnosis of MDD. The K-SADS-PL is a semi-structured interview that provides severity ratings of symptomatology, and assesses current and lifetime history of most *DSM-IV* compatible psychiatric disorders in children and adolescents. Because undergoing K-SADS-PL interviewing constitutes a considerable time burden for our participants and because in the healthy controls, the presence of any Axis-I diagnosis would have excluded them from the study, we opted to use the computerized DISC, which takes significantly less time to administer than the K-SADS-PL, and had trained research assistants administer the DPS over the telephone to further screen potential HCL participants. Both the DISC and DPS have been used extensively by our group and others to determine the presence of Axis-I disorders in adolescents (Tapert et al., 2007; Yang et al., 2009; Perlman et al., 2012; Bava et al., 2013). The final determination of whether potential HCL participants were suitable for admission into the present study was made at weekly consensus meetings between the study personnel and a board certified child and adolescent psychiatrist (TTY).

Depression symptoms measured with the clinician administered Children’s Depression Rating Scale-Revised (CDRS-R; Poznanski, 1996), and the self-report scale Beck Depression Inventory-II (BDI-II; Beck et al., 1996). Anxiety symptoms were measured with the self-report scale Multidimensional Anxiety Scale for Children (MASC; March et al., 1997). In the initial study (Ho et al., 2015) five of the adolescents with MDD did not provide information on age of depression onset but later in follow-up interviews, four of these five participants provided this information, which is now included in the present study. One HCL did not complete the BDI-II and MASC. Participants who did not complete all assessments were excluded from all analyses involving these measures.

In addition to completing forms on basic demographics and general medical and developmental history, all subjects completed the following within 3 days of scanning: Tanner stage (Tanner, 1962), Hollingshead Four-Factor Index of Socioeconomic Position (Hollingshead, 1975), Wechsler’s Abbreviated Scale of Intelligence Test (WASI; Wechsler, 2008), Edinburgh Handedness Inventory, Customary Drinking and Drug Use Record (Brown et al., 1998), Family Interview for Genetics Studies (Maxwell, 1992), Ishihara Color Plates Test (8 plates, 2005 edition), and Standard Snellen Eye Chart (Hetherington, 1954). Groups were matched on age, gender distribution, ethnicity, pubertal status, socioeconomic status, and general intelligence.

Exclusion criteria for adolescents with MDD included a primary psychiatric diagnosis other than MDD, left-handedness, prepubertal stage (Tanner stage < 3), being color blind or having less than 20/40 correctable vision, any contraindication to MR imaging (e.g., pregnancy, claustrophobia, metallic implants), a full scale IQ score < 75 (as determined by WASI), a serious medical or neurological illness, a learning disability, the use of any medication with effects on the central nervous system within 2 weeks of their scan, substance abuse, evidence of illicit drug use or misuse of prescription drugs, and more than two alcoholic drinks per week currently or within the previous month at the time of scanning (as determined by CDDR). Adolescents serving as HCL for this study were excluded based on the same criteria applied to the MDD group, as well as any current or lifetime Axis I psychiatric disorder or any family history of mood or psychotic disorders in first or second-degree relatives (as determined by FIGS).

### Facial Emotion Identification Task

**FIGURE 1:**
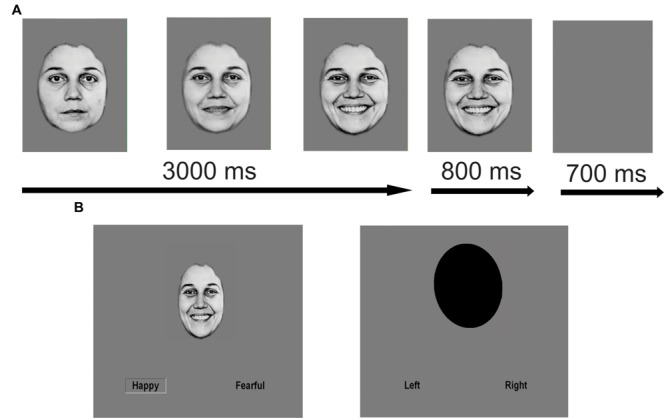
**Schematic of facial emotion identification task.** Facial emotion identification task with dynamically morphing face stimuli. On FACE trials, a screen displaying text of the three possible emotions to discern (FEAR, HAPPY, SAD) was presented for 1500 ms. Next, a neutral face morphed smoothly and dynamically to an emotion of prototypical intensity over the span of 3000 ms. At maximal emotion intensity, the face remained on the screen for an additional 800 ms of the trial before the screen turned blank for 700 ms. At stimulus onset, two possible emotion choices were displayed in text on the bottom left and right corners. Subjects were instructed to press one of two buttons corresponding to the displayed emotion as soon as they recognized the emotion of the face. OVAL trials were used as a sensorimotor control (6 s per trial), where subjects had to determine if the top of an oval was tilting to the left or right and make a button response accordingly as soon as they recognized the tilt direction. **(A)** Example FACE stimulus (enlarged for purposes of clarity). **(B)** Sample FACE and OVAL trial. Reproduced with permission.

As described previously (Ho et al., 2015), we employed an emotion identification task using dynamic morphing face stimuli, which was created and presented using an in-house Tcl script (http://www.tcl.tk/software/tcltk). In this block design task, 10 standardized faces (five female) expressing fear, happiness, and sadness were morphed with computer graphical manipulation. On FACE trials, a screen displaying text of the possible emotions to discern (FEAR, HAPPY, SAD) was presented for 1500 ms. Next, a neutral face morphed smoothly to an emotion of prototypical intensity over the span of 3000 ms and remained on screen for an additional 800 ms before the screen turned blank for 700 ms. At stimulus onset, two possible emotion choices were displayed in the bottom left and right corners; subjects were instructed to press one of two buttons corresponding to their choice as soon as they recognized the facial emotion. OVAL trials (6 s per trial), where subjects had to determine if a morphing oval was tilting left or right (maximal tilt angle = 10°), were used as a sensorimotor control. At the end of the scan, a blank screen was presented for 10 s. One run contained 80 trials (60 FACE trials and 20 OVAL trials) and lasted 490 s in total. RT and accuracy were recorded for each trial. The order of emotion presentation was counterbalanced but not randomized. See **Figure 1** for an illustration of the task.

### LBA Parameter Estimation

Behavioral data from the emotion identification task were modeled using the Linear Ballistic Accumulator (LBA), which is a simplified version of the ballistic accumulator model and the leaky competing accumulator model (Usher and McClelland, 2001; Brown and Heathcote, 2008). The 5 parameters of the LBA model are: (1) *drift rate* (which corresponds to the rate of sensory evidence accumulation or perceptual processing efficiency of the participant); (2) *standard deviation* (which corresponds to how much drift rates can vary across trials); (3) *starting point* (which corresponds to the starting evidence before the decision process begins); (4) *response threshold* (how much evidence is needed before making a choice); and (5) *non-decision time* (time unrelated to the decision process, such as sensory processing or response execution). Changing these parameters changes the model’s predictions of a given individual’s accuracy and RT. For example, larger response thresholds reflect increases in accuracy that is accompanied by both slower responses and more variability in RT. Larger drift rates also reflect increased accuracy but both faster and less variable RT. Non-decision time affects mean RT but has no effect on accuracy or RT variability. The best-fitting LBA parameters, which yield the most adequate match between model predictions and the observed data, can be estimated using a variety of methods, e.g., Bayesian estimation, maximum likelihood estimation, etc.

**FIGURE 2:**
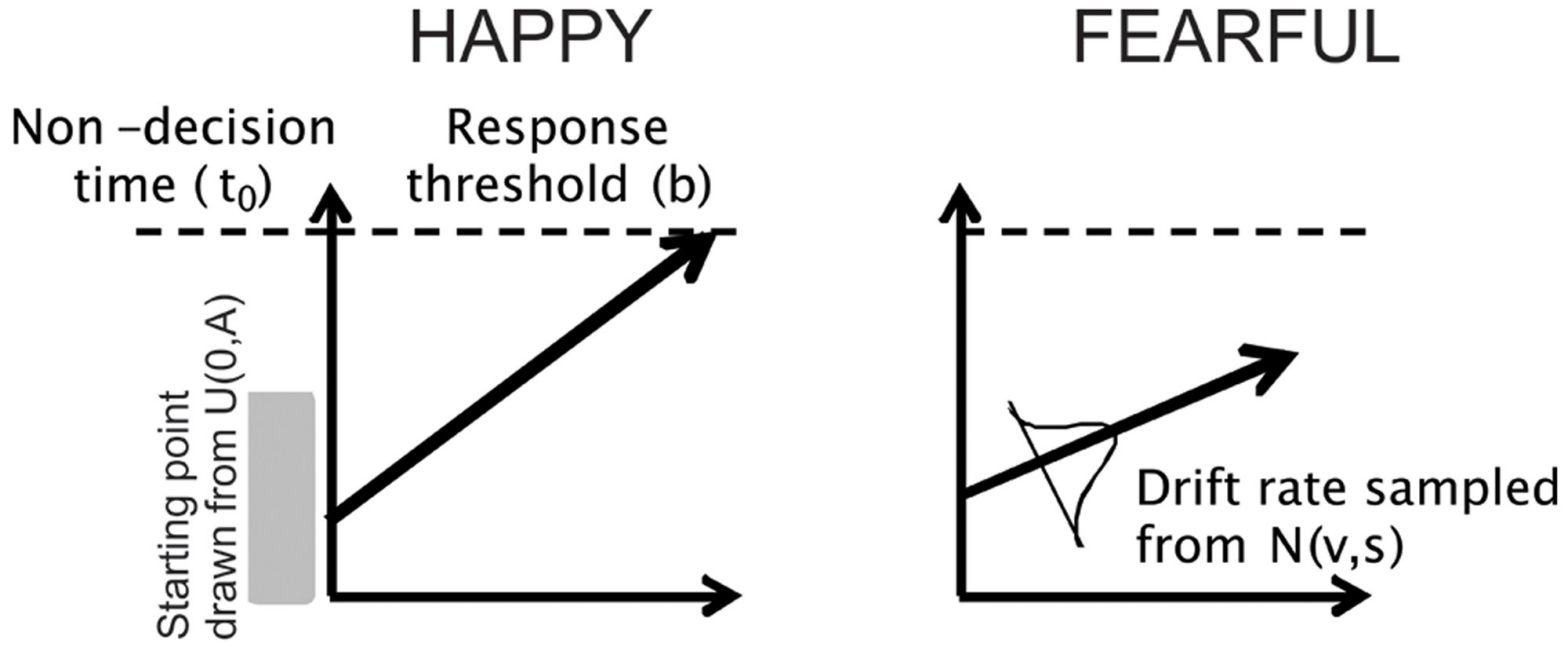
**Schematic of the LBA model.** The 5 parameters of the model are: (1) *drift rate* (which corresponds to the rate of sensory evidence accumulation or perceptual processing efficiency of the participant); (2) *standard deviation* (which corresponds to how much drift rates can vary across trials); (3) *starting point* (starting amount of evidence before the decision process begins); (4) *response threshold* (how much evidence is needed before making a choice); and (5) *non-decision time* (time unrelated to the decision process, such as sensory processing or response execution). The two racing accumulators begin with a random activation level (the starting point) that is independently drawn from a uniform distribution (indicated by the shaded gray area) on [0, A], where *A* is a free model parameter. Activity in each accumulator increases linearly, and a response is triggered as soon as one accumulator reaches the response threshold (*b*). Time is indicated on the abscissa and so the predicted RT is the time taken to reach the threshold, plus a constant offset that represents time unrelated to the decision process (non-decision time, t_0_). The rate at which activation increases in each accumulator is termed the drift rate (*v*). On each trial, the drift rates are drawn from two independent normal distributions (*v, s*). In this example, a subject may need to decide if a face is HAPPY or FEARFUL. The LBA models this two-choice perceptual decision as a race between two “accumulators” that accrue sensory evidence in favor of each choice over time. The first accumulator to gather the criterion amount of evidence determines the subject’s choice and RT (equivalent to the time taken for the accumulator to hit the response threshold plus non-decision time to account for sensory and motor processing time). In the example shown here, the accumulator for “HAPPY” hits the response threshold first, thereby the model predicts the perceptual decision to be a happy face. For more details, see Brown and Heathcote (2008).

In the context of the facial emotion identification task used in this study, on a particular trial a subject may need to decide if a face is HAPPY or FEARFUL. The LBA models this two-choice perceptual decision as a race between two “accumulators” that accrue sensory evidence in favor of each choice over time (with each accumulator representing a perceptual choice, e.g., HAPPY). The two racing accumulators begin with a random activation level (the starting point) that is independently drawn from a uniform distribution on [0, A], where *A* is a free model parameter. Activity in each accumulator increases linearly, and a response is triggered as soon as one accumulator reaches the response threshold (*b*). The predicted RT is the time taken to reach the threshold, plus a constant offset that represents time unrelated to the decision process (non-decision time, t_0_). The rate at which activation increases in each accumulator is termed the drift rate (*v*) for that accumulator which is drawn from a normal distribution (*v, s*). Here, we included the drift rate for the accumulator corresponding to the correct response (termed *perceptual processing efficiency, v_c_*) and drift rate for the accumulator corresponding to the incorrect response (*v_e_*), as we have typically done (Ho et al., 2009, 2012, 2014). On each trial, the drift rates are drawn from two independent normal distributions, with one associated with the correct choice and the other associated with the incorrect choice, with the standard deviations being arbitrarily fixed at 1, as is commonly done in the literature (Forstmann et al., 2008, 2011; Ho et al., 2009, 2012; Mansfield et al., 2011). Hence, the means of the normal distributions are interpreted to reflect the quality or strength of the perceptual input for that particular choice (e.g., FEAR, HAPPY). The first accumulator to gather the criterion amount of evidence determines the subject’s choice and RT (equivalent to the time taken for the accumulator to hit the response threshold plus non-decision time to account for sensory and motor processing time). See **Figure 2** for a conceptual illustration of the LBA and Brown and Heathcote (2008) for more details.

A hierarchical method was used to estimate parameters in the LBA at the individual-level and group-level. We obtained parameter estimates at the individual-level so that we could relate individual differences in aspects of cognitive processing (here, *drift rate*) to individual differences in brain function or clinical characteristics. At the same time, we obtained parameter estimates at the group-level so that we could increase the generalizability of our results and more precisely compute any potential group differences (Turner et al., 2013b; Ho et al., 2014). The hierarchical model makes a key assumption that there are continuous individual differences between people in the parameterization of the cognitive process they use, and the smooth variation of the individual differences is constrained by some central tendency. The group-level analysis estimates the distribution of the individual-level parameters within the population of interest (MDD or HCL), termed a *hyper distribution* (with its own parameters such as the mean, *μ*, and the variance, *σ*). Each individual subject’s data are described by the five parameters of LBA, and these individual parameters, together with the hyper-parameters for their group distributions, are estimated simultaneously using Bayesian posterior sampling methods.

We also made the assumption that depressed compared to healthy control individuals have qualitatively different types of cognitive processes, so we applied the same hierarchical framework to estimate the individual- and group-level parameters for the two groups separately. All hyper distributions were assumed to be truncated normal distributions (truncated to positive values), defined by a mean (*μ*) and standard deviation (*σ*), and were computed separate for each group (MDD, HCL). All individual parameters were fixed across trial conditions except for drift rates (*v_c_* and *v_e_*), which varied across FACE and OVAL trials. Due to the limited number of trials in the facial emotion identification task, all emotion conditions were collapsed together (i.e., FACE trials). Using differential evolution Markov Chain Monte Carlo (DE-MCM) sampling (20 chains, 5000 samples each), we obtained full posterior distributions for each of the 5 LBA parameters (Turner et al., 2013b). DE-MCMC uses multiple interacting chains to generate the *proposal* (a candidate state to be accepted or rejected depending on the acceptance rule) for each sampling step, rather than simply adding random noise to the current state as done by the conventional MCMC. DE-MCMC has proven to be more efficient than the conventional MCMC when the model parameters are highly correlated, as in many sophisticated models of RT (Turner et al., 2013b). For more information on DE-MCMC, please see (Turner et al., 2013b). The individual-level parameters reported here and the ones used in our correlation analysis (see below) are the median of the posterior distributions estimated.

### Group Differences in Drift Rate

An odds ratio (OR) was used to compare MDD and HCL on group-level estimates of drift rate. To compute OR, for each group we compared samples exhaustively drawn from the true distribution. A count was produced reflecting when the value drawn from the MDD distribution was larger than the value drawn from the HCL distribution. The mean count was then divided by 1 minus this count; the OR was therefore calculated to be greater than 1, for ease of interpretation (Ho et al., 2014).

### MR Image Acquisition and Analysis

All scanning was carried out on a GE 3T MR750 System (General Electric Healthcare, Milwaukee, WI, USA) with Twin Speed Gradients and a GE 8-channel head coil at the Center of Functional MRI at the University of California, San Diego. A fast spoiled gradient recalled sequence was used to collect T1-weighted images: TR = 8.1 ms, TE = 3.17 ms, TI = 450 ms, flip angle = 12°, 256 × 256 matrix, FOV = 250 mm × 250 mm, 168 sagittal slices 1 mm thick with an in-plane resolution of 0.98 mm × 0.98 mm. For the facial emotion identification task, T2*-weighted echo planar images (EPI) were acquired using the following pulse sequence: TR = 1000 ms, TE = 30 ms, flip angle = 90°, 64 × 64 matrix, FOV = 192 mm × 192 mm, 490 repetitions, 20 contiguous axial slices 3 mm thick with an in-plane resolution of 3 mm × 3 mm. Participants were supine in the bore of the magnet during the task, and were instructed to relax but be as still as possible while making responses on a button box. Visual stimuli were projected onto a screen and viewed through a small, angled mirror mounted above the participant’s head.

All image processing and analyses were conducted using Analysis of Functional NeuroImages (AFNI; Cox, 1996) and FMRIB Software Library (FSL; Smith et al., 2004). The T1-weighted images were skull-stripped and transformed to MNI152 (Montreal Neurological Institute, Montreal, QC, Canada) with an affine transform (Jenkinson and Smith, 2001; Jenkinson et al., 2002) followed by non-linear refinement (Andersson et al., 2007). Echo planar imaging (EPI) data were slice time and motion corrected and aligned to the T1-weighted images using a localized Pearson correlation function (Saad et al., 2009). Next, the EPI data were convolved with a 4.2-mm full width at half maximum isotropic Gaussian filter and grand mean scaled before being transformed to MNI152 space at 3 mm × 3 mm × 3 mm resolution. Each voxel’s time series was fit using a generalized least squares regression model that estimated the serial correlation of noise using an autoregressive moving average method. Each stimulus type was included as a regressor of interest (FEAR, HAPPY, SAD, OVAL). Each time series of interest spanned stimulus onset until the first valid (≥150 ms) response, before being convolved with a gamma-variate function (Boynton et al., 1996). Demeaned motion parameters and a second-order Legrendre polynomial were included as nuisance regressors (i.e., baseline). Volumes where the Euclidean norm of the motion derivatives were >0.2 or where more than 10% of voxels exceeded the median absolute deviation of the detrended time series were censored (Ho et al., 2015). The mean ± SEM percentage of volumes censored in the MDD group was 5.26% ± 1.06 and in the HCL was 4.84% ± 0.75%. The groups did not differ in the number of volumes censored due to excessive motion (*U* = 509, *p* = 0.903). A general linear test for FACE-OVAL was computed for each participant. Brain activation was operationally defined as percentage signal change relative to baseline.

### Group Differences in Brain Activation to Facial Emotion Identification Task

As described previously (Ho et al., 2015), we assessed group differences on the facial emotion identification task using a linear mixed effects (LME) model on the estimates from the regression model described above, with group (MDD, HCL) modeled as fixed factors and participant modeled as a random factor.

### Correcting For Multiple Comparisons

As described previously (Ho et al., 2015), we empirically derived the minimum number of contiguous voxels (i.e., cluster) using 10,000 iterations of Monte Carlo simulations based on the imposed FWHM values and an average skull-stripped whole brain gray matter mask comprising 24,511 voxels (661,797 μL) that overlapped with at least 90% of the slice stacks created from all participants (Forman et al., 1995). Each voxel in the cluster passed a voxel-wise threshold for a significant group difference at *p* < 0.05. Cluster formation was based on first-nearest neighbor clustering (i.e., voxel faces touching). Cluster correction was conducted at the whole brain level. The empirically derived minimum cluster threshold was 51 voxels (1377 μL).

### Correlation Analysis

Although our *a priori* hypotheses focused specifically on the relationship between perceptual processing efficiency and sensory regions involved in facial emotion processing (e.g., fusiform gyrus, middle occipital cortex), we also correlated perceptual processing efficiency with task activation in all the other regions showing group differences on the task within each group separately. We also conducted exploratory correlations within the MDD group only between perceptual processing efficiency and clinical characteristics (i.e., depression symptom severity as measured by RADS-2 total *t*-scores, anxiety symptom severity as measured by MASC *t*-scores, age of depression onset rounded to the nearest integer year). Finally, we also conducted exploratory correlations within the MDD group only, between activation on each brain region showing group differences on the task and clinical characteristics. All correlations were two-tailed tests using the non-parametric Spearman’s rank correlation coefficient (*r_s_*).

## Results

### Sociodemographic and Clinical Results

As reported previously (Ho et al., 2015), MDD and HCL adolescents did not differ significantly in age, gender, pubertal stage, ethnicity, general intelligence or socioeconomic status (all *p*’s > 0.36). As expected, adolescents with MDD endorsed significantly greater levels of depression and anxiety (all *p*’s < 0.001). See **Table 1** for a summary of the sociodemographic and clinical results.

**Table 1:** Summary of sociodemographic, clinical, and behavioral information of participants.

Characteristic	MDD	HCL	Statistic	*p*-value
Number of participants	26	37		
Gender (M/F)	7/19	14/23	χ^2^ = 0.819	0.366
Age at time of scan (Years)	16.1 ± 0.3	16.0 ± 0.2	t_61_ = 0.29	0.77
Ethnicity (Asian/Black/Caucasian/Hispanic/Mixed)	3/3/8/10/2	4/2/13/14/4	*U =* 9	0.458
Hollingshead socioeconomic score	40 ± 25.2	29 ± 16.3	*U =* 432	0.36
Tanner Score	4.2 ± 0.4	4.0 ± 0.7	*U =* 509	0.90
Wechsler Abbreviated Scale of Intelligence (Standardized)	104.2 ± 4.6	107.3 ± 3.3	*t*_61_ = 0.56	0.58
Beck Depression Inventory II (BDI-II)	28.4 ± 2.0	3.4 ± 0.7	t_60_ = 13.10	<0.0001
Children’s Depression Rating Scale– Revised (Standardized)	73.1 ± 1.8	34.3 ± 1.2	t_61_ = 18.64	<0.0001
Multidimensional Anxiety Scale for Children (Standardized)	59.8 ± 1.8	42.1 ± 1.4	t_60_ = 7.88	<0.0001
Mean response time (seconds)	2.25 ± 2.16	2.16 ± 0.59	t_61_ = 0.53	0.958
Mean accuracy (%)	80.73 ± 6.44	80.06 ± 9.73	t_61_ = 0.11	0.913
Age of depression onset (years)	12.34 ± 0.57			

*All entries are of the form mean ± SEM. Statistical analyses of between-group differences were conducted with χ^2^, Student’s *t*-tests, and Wilcoxon rank sum test (*U*). All *p*-values indicate two-tailed statistical significance levels.*

### Behavioral Results

The MDD and HCL groups did not differ significantly in mean accuracy or mean RT on the task (see **Table 1**). A two-way ANOVA with group (MDD, HCL) and emotion (FEAR, HAPPY, SAD) as factors were applied to the accuracy and RT data separately. There was no main effect of group or emotion on accuracy (all *p*’s > 0.5). For RT, there was no main effect of group (*F*_1,198_ = 0.284, *p* = 0.595), a significant effect of emotion where participants were significantly faster on HAPPY trials (*F*_2,198_ = 7.328, *p* < 0.001), but no significant group × emotion interaction (*F*_2,198_ = 0,150, *p* = 0.861).

### LBA Results

**FIGURE 3:**
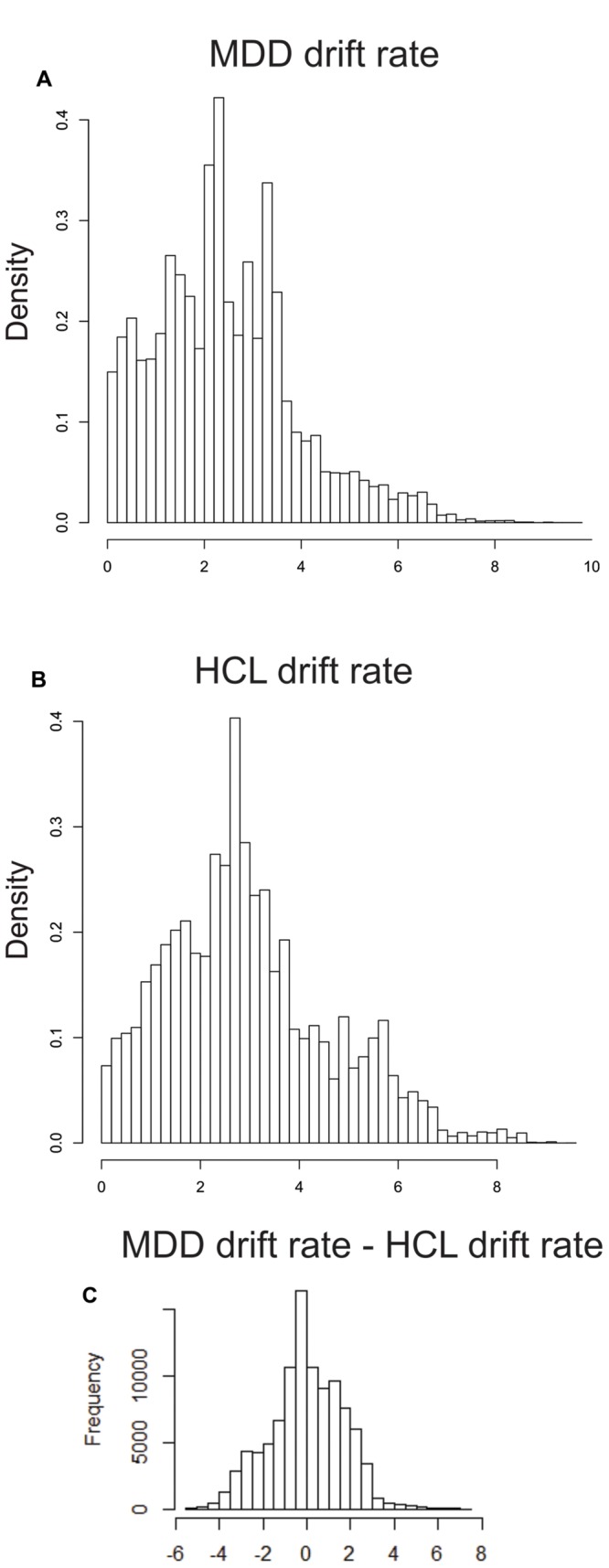
**Group differences in posterior estimates of drift rate.** We used the mean (μ) and variability (*σ*) hyper distributions for drift rate on correct trials (*v_c_*) to calculate the mean of the associated truncated normal distribution for MDD **(A)** and **(B)** HCL. **(C)** Differences in these means (MDD – HCL). See *LBA Parameter Estimation* in the *Methods* for more details.

MDD and HCL did not differ significantly in group-level estimates of drift rate (OR = 1.58:1), not providing support of the MDD group exhibiting greater drift rates than the HCL group. **Figure 3** shows the hyper distribution of the drift rate parameter of interest (*v_c_*) for each group separately as well as the group-level difference (MDD-HCL). This difference distribution is centered on 0, thereby indicating no group difference in drift rate.

### Group Differences in Brain Activation on Facial Emotion Identification Task

**FIGURE 4:**
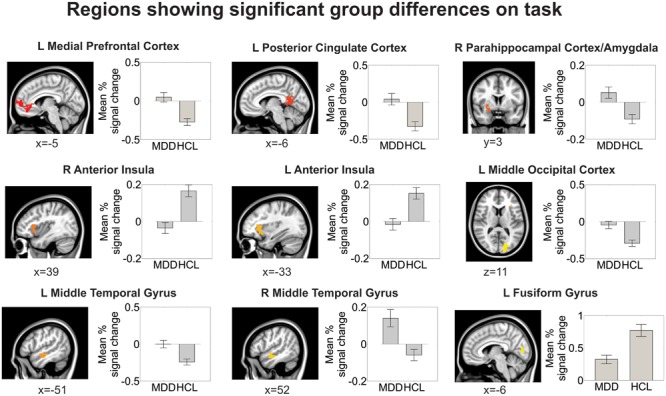
**Group differences in brain activation during the facial emotion identification task.** All regions shown here are corrected for multiple comparisons at a cluster-wise threshold of *p* < 0.05 (see *Materials and Methods* for more details). Locations are reported in Montreal Neurological coordinates (radiological convention).

Relative to the HCL group, adolescents with MDD showed hyperactivation in the left medial prefrontal cortex and left posterior cingulate cortex, as reported previously (Ho et al., 2015). Relative to the HCL group, adolescents with MDD also showed hypoactivation in bilateral anterior insula and left fusiform gyrus/lingual gyrus, as well as hyperactivation in a cluster encompassing right parahippocampal cortex, amygdala, and lentiform nucleus, hyperactivation in bilateral middle temporal gyri, and hyperactivation in left middle occipital cortex. See **Figure 4** and **Table 2** for more details.

**Table 2:** Summary of location and size of brain regions showing significant group differences on facial emotion identification task.

Region	MDD	HCL	Location (x,y,z)	# of voxels
L medial prefrontal cortex, pregenual cingulate cortex	0.055 ± 0.07	-0.31 ± 0.05	-4, -51, -2	350
L posterior cingulate cortex	0.087 ± 0.09	-0.296 ± 0.06	-2, -51, 15	189
R parahippocampal cortex, amygdala, lentiform nucleus	0.049 ± 0.03	-0.109 ± 0.03	27, -7, 5	170
R anterior insula, inferior frontal gyrus	-0.043 ± 0.03	0.168 ± 0.03	34, 23, 2	140
L anterior insula, inferior frontal gyrus	-0.038 ± 0.03	0.147 ± 0.03	-33, 21, 0	130
R middle temporal gyrus	0.109 ± 0.05	-0.086 ± 0.03	54, -6, -13	116
L middle temporal gyrus	0.007 ± 0.06	-0.231 ± 0.04	-56, -13, -10	91
L fusiform gyrus, lingual gyrus	0.321 ± 0.06	0.77 ± 0.09	-22, -88, -10	69
L middle occipital cortex	-0.025 ± 0.07	-0.304 ± 0.05	-42, -78, 6	57

*Brain activation is reported as mean ± SEM of percentage signal change on FACE-OVAL for each group (MDD, HCL). All results are corrected for multiple comparisons at a cluster-wise threshold of *p* < 0.05 (see *Materials and Methods* for more details). Locations are reported according to the center of mass of cluster in Montreal Neurological coordinates (radiological convention).*

### Correlations Between Perceptual Processing Efficiency and Brain Activation on Facial Emotion Identification Task

**FIGURE 5:**
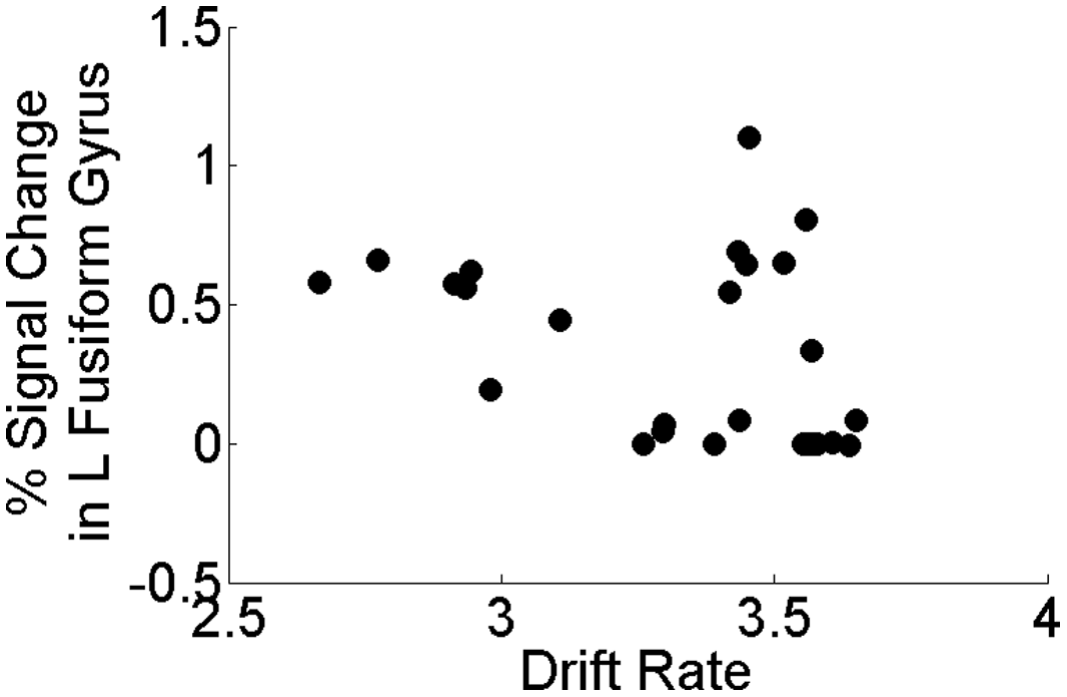
**Correlation between drift rate (*v*_c_) and brain activation in left fusiform gyrus within the MDD group only.** Perceptual processing efficiency was negatively associated with task activation in left fusiform gyrus (*r_s_* = -0.441, *p* = 0.021) in the MDD group only. All correlations were two-tailed tests using the non-parametric Spearman’s rank correlation coefficient.

Our *a priori* hypotheses concerned relating perceptual processing efficiency (*v*_c_) to task activation from sensory regions that have been previously demonstrated to show differences between depressed and healthy individuals (e.g., fusiform gyrus, occipital cortex). In the present study, we found that within the MDD group only, perceptual processing efficiency was negatively associated with task activation in left fusiform gyrus (*r_s_* = -0.441, *p* = 0.021; **Figure 5**) but was not significantly associated with task activation in left middle occipital cortex (*p* = 0.325). All other brain regions showing group differences on the task did not correlate significantly with perceptual processing efficiency within the MDD group (all *p*’s > 0.487). Importantly, task activation of left fusiform gyrus did not correlate with mean RT or accuracy on the task within the MDD (all *p*’s > 0.17). Finally, within the HCL, none of the brain regions showing group differences on the task correlated significantly with perceptual processing efficiency, mean RT, or mean accuracy (*p*’s > 0.123).

### Additional Exploratory Correlations

We also conducted additional exploratory correlations within the MDD group only between clinical characteristics (depression symptom severity, anxiety symptom severity, and age of depression onset) and (1) perceptual processing efficiency and (2) task activation on all other brain regions showing between group differences. All of these relationships were non-significant (all *p*’s > 0.07).

## Discussion

This is the largest study to date to combine mathematical models of choice behavior with fMRI activation for improved understanding of cognitive and neural mechanisms of adolescent MDD. We examined the relationship between perceptual processing efficiency—as ascertained from RT distributions subjected to a cognitive-behavioral model of RT—and brain activation in adolescents with MDD and well-matched healthy controls (HCL) during performance of a facial emotion identification task. The advantage of using a mathematical model of choice behavior over traditional analyses of accuracy and RT is that distinct components of the decision making process can be assessed. This method therefore allows us to determine which cognitive components or processes altered under the influence of a task condition, or as in this case, psychopathology of the individual. Here, motivated by prior work in behavioral studies of adults (Pe et al., 2013) and adolescents (Ho et al., 2014) with MDD, we used the LBA model (Brown and Heathcote, 2008) and estimated individual-level and group-level estimates of the *drift rate* parameter as a proxy of perceptual processing efficiency. We found that while adolescents with MDD and HCL did not differ in group-level estimates of perceptual processing efficiency (**Figure 3**), adolescents with MDD exhibited abnormal activation to emotional faces throughout the face processing network, including early visual processing regions, limbic and paralimbic regions, and top–down frontal regions (**Figure 4**). Notably, adolescents with MDD exhibited hypoactivation relative to HCL in a key face processing area, the left fusiform gyrus. Moreover, within the MDD group only, reduced left fusiform gyrus activation to emotional faces was significantly associated with greater individual-level estimates of perceptual processing efficiency (**Figure 5**). Importantly, activation in left fusiform gyrus did not correlate with mean RT or mean accuracy on the task, demonstrating the utility of combining cognitive models of behavior with neuroimaging methods to better understand neural correlates of cognitive mechanisms in clinical populations. Together, our results suggest that affective processing biases in adolescents with MDD are characterized by greater perceptual processing efficiency of affective visual information in sensory brain regions responsible for processing of visual information.

Several studies in both adult and adolescent depression have documented functional and structural differences in visual regions such as the fusiform/lingual gyrus and middle occipital cortex (Ho et al., 2013, 2014; Liao et al., 2013; Truong et al., 2013; Henje Blom et al., 2015). The results from the present study are consistent with the findings of fusiform gyrus dysfunction in depression and particularly, with recent fMRI studies reporting reduced fusiform gyrus activation during affective face processing in adolescents with MDD. For instance, in a study where participants judged happy, sad, fearful, and neutral faces, adolescents with MDD exhibited reduced fusiform gyrus activation across most valence contrasts and especially during processing of happy versus sad face stimuli (Henje Blom et al., 2015). Similarly, in another study where participants judged fearful faces of varying intensities, adolescents with MDD showed comparatively reduced functional connectivity between the fusiform gyrus and the subgenual anterior cingulate cortex, a key region interfacing between emotional and cognitive stimuli processing (Ho et al., 2014). Importantly, our results build from our previous investigation of the same data set (where we examined task-based and resting-state functional connectivity of the primary nodes of a major task-negative network) by demonstrating the importance of sensory regions (and by extension, networks) that may affect processing of sensory stimuli and consequently, support the cognitive processing biases found in MDD (Ho et al., 2015). By innovatively combining neuroimaging and mathematical models of choice behavior, the present study suggests that reduced fusiform gyrus activation in adolescents with MDD reflects more efficient perceptual processing of affectively laden stimuli and highlights the importance of examining sensory regions—such as occipital cortex—to better understand cognitive processing in MDD.

Compellingly, research has also shown that activation in occipital regions to emotional faces predicts antidepressive response in individuals with MDD (Surguladze et al., 2005; Keedwell et al., 2009, 2010; Furey et al., 2013, 2015). MDD is characterized by an affective processing bias, wherein processing of emotional information (such as facial expressions) is biased toward negative and away from positive or neutral information (Gotlib et al., 2004; Foland-Ross and Gotlib, 2012). This affective processing bias can be interpreted within the framework of sensory processing mechanisms in visual cortices and more specifically, the cholinergic system (Furey, 2011). Specifically, cholinergic dysfunction in MDD may lead to selectively improved (or more efficient) processing of emotional features in visual stimuli (Vuilleumier and Driver, 2007; Bentley et al., 2011; Furey, 2011). Indeed, in healthy individuals, the cholinergic system differentially modulates responses to stimuli in visual processing areas of the brain based on emotional content (Vuilleumier and Driver, 2007; Bentley et al., 2011). In the present study, we observed reduced fusiform gyrus activation to emotional faces in adolescents with MDD relative to HCL, which may reflect greater perceptual processing, as evidenced by a significant inverse correlation between fusiform gyrus activation on the task and drift rate estimates. Consistent with our present findings, one study reported that adults with MDD exhibited reduced activation in occipital cortex relative to healthy controls during emotional processing of face stimuli in a working memory task (Furey et al., 2013). Interestingly, this study also demonstrated that activation in occipital regions that responded selectively to emotional content of visual stimuli correlated with treatment responses to the anticholinergic antidepressant scopolamine. Greater improvement in depressive symptoms was also associated with greater change in activation of occipital cortex to emotional stimuli after scopolamine administration relative to baseline (Furey et al., 2013). In another study, adults with MDD exhibiting greater activation in middle occipital cortex to sad versus happy facial expressions at baseline showed larger clinical responses to scopolamine (Furey et al., 2015). While currently speculative, this hypothesized link between cholinergic dysfunction, processing biases of affective stimuli, and differential response to affective stimuli in visual areas may explain why we found that abnormally reduced fusiform gyrus activation was significantly associated with greater perceptual processing efficiency to emotional faces in adolescents with MDD.

Nevertheless, the results from this study must be interpreted in light of its limitations. Firstly, the task utilized in this study was not optimally designed to have the behavioral data modeled by the LBA, which may explain why group-level differences in drift rate were not observed in our sample. It is also possible that the facial emotion identification task used in the present study measures more than perceptual processing of affect. However, given the straightforward requirements of the task and the fact that prior studies using sequential sampling models like the LBA to investigate individuals with depression focused on the drift rate, we believed the drift rate was the most appropriate parameter from the LBA to investigate in the present study. Nevertheless, future studies could utilize tasks that evoke a wider range of individual variability on task performance so that enough trials comprise the incorrect RT distributions to permit better modeling of these data. Future studies could also include more cognitively challenging tasks and could manipulate the response window to affect difficulty and other relevant parameters in the LBA (e.g., response bias, non-decision time). Another limitation to our study design is that the presentation of emotion conditions was not randomized across subjects. Future studies should increase the number of trials for each emotion condition so that drift rates (or other relevant model parameters) can be sufficiently estimated for each emotion condition. Increasing the number of trials per condition will also provide the necessary power to investigate negative versus positive information processing biases in this population.

Secondly, we relied on the CDDR to measure drug and alcohol consumption behaviors from our study participants, which assesses age of first and regular use as well as a general use pattern for alcohol, nicotine, and other drugs since age 13. The CDDR is interview administered and has strong internal consistency and validity (Brown et al., 1998). Nevertheless, future studies recruiting adolescents from the community or from outpatient clinics should employ objective tests to ensure that drug and alcohol use are not potential fMRI confounds.

Thirdly, our sample of depressed adolescents included individuals with comorbid psychiatric diagnoses. With the exception of one subject (i.e., ADHD), all of the comorbid conditions present in our cohort of depressed adolescents were anxiety disorders. The rate of comorbid anxiety disorders in our depressed sample (61.5%) matches the rate at which adolescent depression presents with comorbid anxiety in the general community, which is estimated to be around 60% (Essau, 2008; Kessler et al., 2012). Our depressed adolescent sample thus reflects the distribution of anxiety disorders in the population of adolescents with depression typically seen in outpatient clinics which increases the generalizability of our findings. Moreover, we did not find any correlations between our self-report measure of anxiety with perceptual processing efficiency or brain activation in regions showing group differences on the task, suggesting that our results are not driven by anxiety. Nevertheless, future studies combining models such as the LBA with neuroimaging in individuals with depression and comorbid anxiety are needed to test if our results are specific to depression or if fusiform gyrus dysfunction and its relationship to perceptual processing efficiency represent a transdiagnostic dimension of emotional dysregulation (Insel et al., 2010; Sanislow et al., 2010; Cuthbert and Insel, 2013).

Finally, the cross-sectional design of the present study limits us from investigating whether fusiform gyrus dysfunction to emotional information is a trait or state marker of adolescent MDD. Longitudinal studies are needed to determine if greater perceptual processing efficiency and reduced fusiform gyrus activation to emotional stimuli are potential risk factors for developing MDD. Similarly, longitudinal work is needed to determine if drift rates, fusiform activation, and the association between these two measures could potentially serve as markers for treatment response. Adolescence is a time of ongoing maturation of neural networks, including visual systems (Power et al., 2010; Chai et al., 2014) and future longitudinal studies may employ functional connectivity analyses to relate developmental changes in visual networks, perceptual processing efficiency to emotional stimuli, and depressive symptomatology.

In summary, combining models linking behavior to cognition, such as RT models, in conjunction with fMRI provide informative insight into both basic cognitive processes (Ho et al., 2009, 2012; van Maanen et al., 2011; Mulder et al., 2012) and cognitive processing altered by neurological or psychiatric insult (Ho et al., 2014). The present study is the largest to date to utilize such methods to better understand emotional processing in depression. Our results clarify the neural correlates of affective biases commonly observed in this patient population by demonstrating that perceptual processing efficiency to emotional stimuli is elevated in depressed adolescents and accompanied by dysfunction in early sensory processing regions. Our results present intriguing new hypotheses to test, including whether early sensory processing regions relevant to task demands (e.g., fusiform gyrus, middle occipital cortex) are related to depression onset and if onset of depression affects the brain development of intrinsic functional networks, including the visual system. The development of novel models that combine accuracy, RT, and fMRI responses into a single unified model will help bridge the gap between our abstract understanding of cognitive processing and the signals derived from brain imaging data, thereby allowing researchers to explicitly test specific cognitive theories as well as to ensure that the cognitive abstractions assumed are biologically plausible (Turner et al., 2013a, 2015).

## Author Contributions

TCH designed the experiment, analyzed the data, created the figures, and wrote the paper. SZ analyzed the data and assisted with manuscript preparation. All other authors assisted with manuscript preparation and approved the final version for publication.

## Conflict of Interest Statement

The authors declare that the research was conducted in the absence of any commercial or financial relationships that could be construed as a potential conflict of interest.
